# Mind Over Menopause: Bridging the Gap in Mental Health Care

**DOI:** 10.1192/bjo.2024.475

**Published:** 2024-08-01

**Authors:** Alexandra Thatcher, Catherine Graham, Grace Denton, Katherine Kearly-Shiers, Kristyn Manley

**Affiliations:** 1 Gloucestershire Health and Care NHS Trust, Cheltenham, United Kingdom; 2 Avon and Wiltshire Partnership NHS Trust, Bristol, United Kingdom; 3 University Hospitals Bristol and Weston NHS Trust, Bristol, United Kingdom

## Abstract

**Aims:**

The effect of menopause on mental health is increasingly well recognised. Studies assessing peri- and post-menopausal women report higher incidences of depression and anxiety. Without recognition and treatment, the negative impact on mental health during menopause can lead to long-lasting effects on quality of life. NICE and the British Menopause Society (BMS) guidelines recommend cognitive behavioural therapy (CBT) and an individualised approach, for women experiencing depression and anxiety as a result of menopause. The aim of this project was to collect data relating to the provision of mental health interventions (and how they are accessed) for women seeing menopause specialists across the UK. This data can then be used to inform and promote improvements in the delivery of care for menopause mood symptoms.

**Methods:**

An expert panel of psychiatry, gynaecology and general practice clinicians designed an online survey which considered NICE/BMS guidance, the current evidence base and local referral/funding pathways. This was piloted on health care professionals in primary and secondary care before review by BMS Council Members. A link to the survey was distributed via email to members of the BMS on one occasion.

**Results:**

139 responses were received from menopause specialists across the 15 UK Deaneries. 71% worked in primary care and 29% in secondary care. 65% of clinicians offer CBT for mood symptoms but 99% reported suboptimal provision of this intervention. 43% of respondents reported over half of their patients with mood symptoms would benefit from psychological support, however 80% do not have a designated mental health wellbeing practitioner. 35% of specialists have referred complex patients to secondary mental health services. When asked what mental health resources would be most beneficial for their patients, 83% desired improved access to CBT, 65% psychological support attached to all menopause clinics, 53% guidance on managing mood symptoms in menopause and 39% an MDT clinic.

**Conclusion:**

The data suggests that complex mood disorders are common in women presenting to menopause services and require non-hormonal interventions to support benefits seen with HRT. The results suggest poor provision of psychological interventions, particularly talking therapies, for women experiencing mood disorders as part of their menopause. Improved cross-specialty working and training, and improved access to CBT were identified as methods of addressing this. Locally, these results have formed the basis of a service funding bid for CBT and development of a pilot cross-speciality gynaecology/psychiatry MDT Hub.

